# Inherited Glutathione Reductase Deficiency and *Plasmodium falciparum* Malaria—A Case Study

**DOI:** 10.1371/journal.pone.0007303

**Published:** 2009-10-06

**Authors:** Valentina Gallo, Evelin Schwarzer, Stefan Rahlfs, R. Heiner Schirmer, Rob van Zwieten, Dirk Roos, Paolo Arese, Katja Becker

**Affiliations:** 1 Dipartimento di Genetica, Biologia e Biochimica, University of Turin, Turin, Italy; 2 Interdisziplinäres Forschungszentrum, Gießen University, Gießen, Germany; 3 Biochemie-Zentrum Heidelberg, Heidelberg University, Heidelberg, Germany; 4 Sanquin Research and Landsteiner Laboratory, Academic Medical Centre, University of Amsterdam, Amsterdam, The Netherlands; New England Biolabs, United States of America

## Abstract

In *Plasmodium falciparum*-infected red blood cells (RBCs), the flavoenzyme glutathione reductase (GR) regenerates reduced glutathione, which is essential for antioxidant defense. GR utilizes NADPH produced in the pentose phosphate shunt by glucose-6-phosphate dehydrogenase (G6PD). Thus, conditions affecting host G6PD or GR induce increased sensitivity to oxidants. Hereditary G6PD deficiency is frequent in malaria endemic areas and provides protection against severe malaria. Furthermore, GR deficiency resulting from insufficient saturation of the enzyme with its prosthetic group FAD is common. Based on these naturally occurring phenomena, GR of malaria parasites and their host cells represent attractive antimalarial drug targets. Recently we were given the opportunity to examine invasion, growth, and drug sensitivity of three *P. falciparum* strains (3D7, K1, and Palo Alto) in the RBCs from three homozygous individuals with total GR deficiency resulting from mutations in the apoprotein. Invasion or growth in the GR-deficient RBCs was not impaired for any of the parasite strains tested. Drug sensitivity to chloroquine, artemisinin, and methylene blue was comparable to parasites grown in GR-sufficient RBCs and sensitivity towards paraquat and sodium nitroprusside was only slightly enhanced. In contrast, membrane deposition of hemichromes as well as the opsonizing complement C3b fragments and phagocytosis were strongly increased in ring-infected RBCs of the GR-deficient individuals compared to ring-infected normal RBCs. Also, in one of the individuals, membrane-bound autologous IgGs were significantly enhanced. Thus, based on our *in vitro* data, GR deficiency and drug-induced GR inhibition may protect from malaria by inducing enhanced ring stage phagocytosis rather than by impairing parasite growth directly.

## Introduction

The tripeptide glutathione (γ-glutamylcysteinylglycine) is present in millimolar concentrations in the malaria parasite *Plasmodium falciparum* as well as in the host red blood cell (RBC) [1]–[5]. Reduced glutathione (GSH) plays an essential role in antioxidant defense in both parasite and host cell [1]–[5]. Parasite GSH supports cell growth by providing electrons for deoxyribonucleotide synthesis and takes part in detoxifying heme, a product of hemoglobin digestion [6]. Furthermore, GSH is the coenzyme of the glyoxalase system, which detoxifies methylglyoxal [7], and of glutathione *S*-transferase (GST)-based reactions. GSTs contribute to the detoxification of exogenous and endogenous cytotoxic metabolites. In *Plasmodium*, GST is involved in peroxide detoxification and the binding of parasitotoxic heme as well as in the development of drug resistance [8]. The flavoenzyme glutathione reductase (GR, EC 1.8.1.7) reduces oxidized glutathione (GSSG) back to GSH [3], [9]. Due to its central position in redox control, *P. falciparum* GR (*Pf*GR) is ranked number one in the list of proposed antimalarial drug targets for *Plasmodium* (http://tdrtargets.org/), and a wide range of respective drug development approaches is currently being followed [9]–[11]. In addition, the inhibition of RBC GR has been proposed as an approach to reduce the risk of multidrug resistance in malaria parasites [9].

In the GR-catalyzed reaction, reducing equivalents are provided by NADPH. NADPH is generated in the first half of the hexose monophosphate shunt by glucose-6-phosphate dehydrogenase (G6PD). Therefore, G6PD (producer of NADPH) as well as GR (utilizer of NADPH) are equally essential to maintain GSH homeostasis in the parasite-host unit [2], [4]. Mutations affecting either G6PD or GR might thus induce similar metabolic and functional consequences in the RBC. G6PD deficiency occurs in numerous genotypes, some of which are polymorphic and particularly frequent in areas where malaria is or was endemic [12]–[14], affecting approximately 330 million people worldwide [15]. Decreased GR activity due to low saturation with FAD is also common in certain malaria-endemic regions [16]. By contrast, hereditary GR deficiency is rare [17], and only recently a full biochemical and molecular characterization of a GR mutation leading to complete GR deficiency has been performed [18]. In this patient, RBCs and leukocytes did not contain any GR activity, and the GR protein could not be detected by Western blotting. DNA sequencing revealed a 2242-bp deletion, starting at nucleotide +658 in intron 11 and ending at nucleotide 639 in the 3′ untranslated region of exon 13 of the GR gene, which is located on chromosome 8. As a result, translated GR missed the complete dimerization domain, resulting in an inactive enzyme [18].

In view of (a) the potentially similar metabolic effects of G6PD and GR deficiency, (b) the well documented protection from severe *falciparum* malaria afforded by G6PD deficiency [14], [19] and (c) the fact that *P. falciparum* GR and human GR represent most promising antimalarial drug targets, we studied invasion and growth of several *P. falciparum* strains in GR-deficient RBCs as well as the stage-dependent pathological alterations induced by parasite growth in these erythrocytes. We directly compared those changes to GR-sufficient control cells as well as to analogous data obtained with malaria-infected G6PD-deficient RBCs and to senescent RBCs [13], [20], [21]. Analogies with RBCs from patients with sickle-cell trait, β-thalassemia [22], and pyruvate kinase deficiency [23] are discussed.

## Results

Unless otherwise indicated, all experiments reported below were performed with RBCs from the index patient.

### Invasion and multiplication of P. falciparum grown in GR-deficient RBCs

Twenty-four hours after inoculation of GR-deficient RBCs with malarial parasites (strains 3D7 or K1, experiment 1, see Materials and Methods), ring stages of *Plasmodium* were detectable in all wells. As determined by Giemsa staining and by having an experienced technician count infected cells under the light microscope, the parasitemia for the 3D7 strain was 3.9±0.5% in the GR-deficient cells and 4.0±0.4% in the controls. This indicated that *Plasmodium* is able to invade GR-deficient RBCs as efficiently as normal RBCs. Subsequently, parasites were grown in the respective RBC cultures for four complete 48-h cycles, showing a mean multiplication rate of 4.9±0.3 per RBC cycle for the GR-deficient RBCs, as well as 4.3 (0^+^ blood) and 6.5 (A^+^ blood) for the controls (mean 5.4±0.5; data are given in Table 1).

**Table 1:** Comparison of growth and biochemical properties of *P. falciparum* grown in GR-deficient and control RBCs.

	GR-deficient RBCs	Control RBCs
aTotal glutathione in the parasite		
(3D7, exp. 1+2) [nmol/mg protein]	68±2.1	73±1.4
bGR activity in parasites [mU/mg]	160±6	270±14* (P = 0.002)
Parasite multiplication rate per RBC		
cycle (3D7, exp. 1))	4.9±0.3	5.4±0.5
IC_50_ chloroquine (3D7) [nM]	8.2±0.2 (4.9±0.2)	8.0±0.2 (A^+^) (5.9±0.6)
IC_50_ chloroquine (K1) [nM]	160±10	190±11 (A^+^)
IC_50_ methylene blue (3D7) [nM]	3.9±0.2 (4.2±0.3)	3.8±0.2 (A^+^) (4.2±0.3)
IC_50_ methylene blue (K1) [nM]	8.8±0.5	8.1±0.4 (A^+^)
IC_50_ artemisinin (3D7, exp. 2) [nM]	14±0.5	16±0.8
IC_50_ paraquat (3D7, exp. 2) [μM]	42±2	53±3* (P = 0.04)
IC_50_ SNP (3D7, exp. 2) [μM]	5.8±0.3	11±0.8* (P = 0.006)

Experiment 1 refers to the index patient and the A^+^ blood group control, experiment 2 (data given in brackets or indicated as exp. 2) refers to index patient and mean values of 3 controls. All values given represent mean values ± SEM of the different individuals included and two to three parallel determinations per sample.

a Before determining the biochemical parameters in the parasites, *P. falciparum* was grown over 4–5 cycles (corresponding to 8–10 days) in the respective RBCs. Control cells had the same blood group (0^+^) as the patient's.

b *Indicates significant differences (P<0.05) between GR-deficient cells and controls.

Also, in the second experiment (strain 3D7, see Methods), all RBC samples (index patient and three controls) were efficiently invaded by the parasites. Forty-eight hours later about 4% infected RBCs (IRBCs) were again determined in all 4 cultures. After splitting and another 48-h cycle, a very similar parasitemia (given in the following for 3D7) was determined in all samples (9.8±0.3% in the GR-deficient cells, 9.3±0.4% in the controls). Also the percentages of ring stages (36±2% in the patient and 37±3% in the controls) and trophozoite/schizont stages (64±1% in the patient, 62±3% in the controls) did not differ significantly. The morphology of the parasites as determined by light microscopy after Giemsa staining was unchanged throughout the experiments (data not shown). In a separate set of experiments performed in the other participating laboratory in Turin with the Palo Alto parasite strain, similar data were obtained, showing that the invasion rate of GR-deficient RBCs was as high as that of control RBCs (30% parasitemia at 19 h after inoculation with 6.4% schizonts), and regular development into trophozoites was observed on day 2 (30% parasitemia).

### GR activity, total glutathione content and drug sensitivity of P. falciparum grown in GR-deficient RBCs

Malarial parasites grown in GR-deficient RBCs were tested for total glutathione content, GR activity, and their sensitivity towards redox-active antimalarials as well as inducers of oxidative and nitrosative stress. Data were collected in two independent experiments (including venipuncture and shipment of the blood samples) with different controls and two different parasite strains (K1 and 3D7). Before determining the above parameters, parasites were grown for 4–5 cycles (corresponding to 8–10 days) in the respective RBCs. Late trophozoite stages were then isolated as described in the Materials and Methods section and directly used for the determination of GR activity and glutathione content. Antimalarial drugs and pro-oxidant agents were added to the ring stages of the parasites in order to determine their IC_50_ values.

In both experiments the parasites displayed a glutathione content of about 70 nmol/mg protein, which did not differ between parasites grown in GR-deficient and normal host cells (Table 1). However, in the parasites grown in GR-deficient cells, GR activity was found to be significantly reduced to 61% (experiment 1, see Table 1) of control values. A comparable value (66% of control activity) was determined in experiment 2.

The IC_50_ values for CQ, artemisinin, and methylene blue were very similar between parasites grown in GR-deficient RBCs and in normal control RBCs (Table 1). This was verified in both experiments and for both parasite strains K1 and 3D7. However, the IC_50_ values of the superoxide generating agent paraquat and of sodium nitroprusside were found to be significantly lower in parasites grown in GR-deficient RBCs than in controls (Table 1).

### Membrane binding of hemichromes, complement C3 fragment, autologous IgG, and phagocytosis in ring stage-infected GR-deficient RBCs

**Figure 1:**
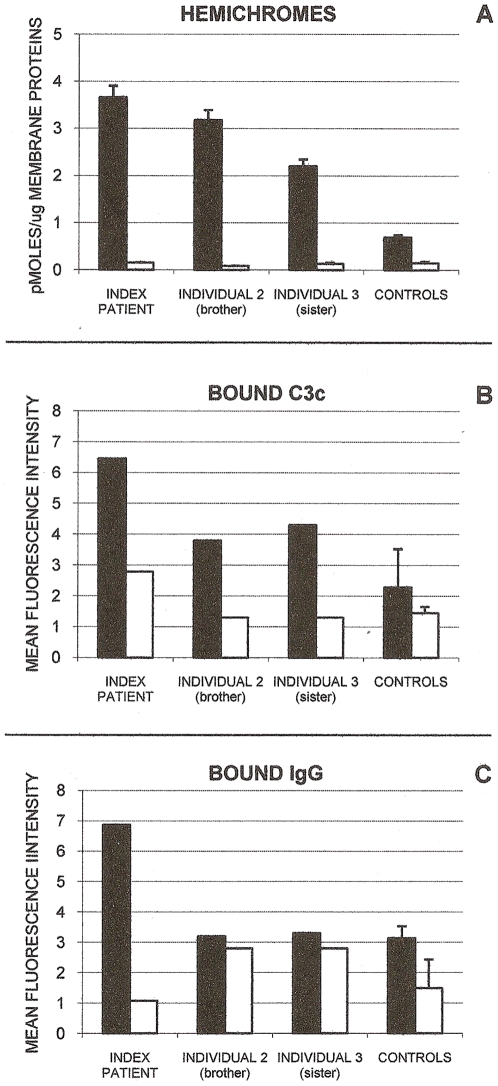
Membrane-bound hemichromes, complement C3c fragment, and autologous IgG in GR-deficient and GR-sufficient RBCs. Shown are membrane-bound hemichromes (panel A), complement C3c fragment (panel B) and autologous IgG (panel C) in ring-infected and non-infected GR-deficient RBCs from the index patient, brother (individual 2) and sister (individual 3) of index patient, and from normal GR-sufficient controls. Black bars indicate ring-infected RBCs; open bars, non-infected RBCs. Hemichromes are expressed as pmoles/µg membrane protein. Mean values of index patient, individuals 2 and 3, and normal controls (mean±SD, n = 2–4) are shown. Hemichromes were significantly higher (p<0.02) in the ring-infected RBCs of the index patient and individuals 2 and 3 compared to ring-infected RBCs of normal controls. Complement C3c fragment and autologous IgG are expressed as Mean Fluorescence Intensity. Representative data of GR-deficient individuals and mean values of normal controls (mean±SD, n = 2–3). For experimental details see Materials and Methods.

Membrane-bound hemichromes, indicators of oxidative membrane damage and inducers of RBC membrane modifications producing enhanced phagocytosis [20]–[22], were measured in ring stage-infected RBCs of the index patient, individual 2 (brother), and individual 3 (sister), all homozygous for the GR deficiency, and were compared to ring stage-infected normal RBCs. As shown in Fig. 1A, parasite growth significantly increased membrane-bound hemichromes in all three GR-deficient individuals.

**Figure 2:**
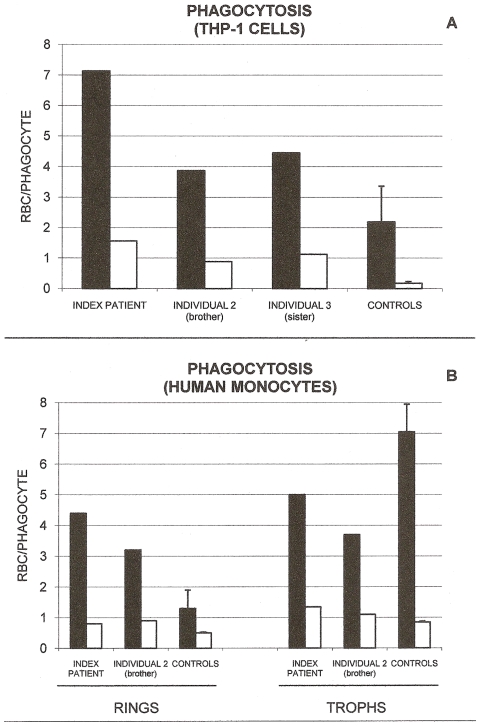
Phagocytosis of GR-deficient and GR-sufficient RBCs. Shown is the phagocytosis by THP-1 cells (panel A) of ring-infected and non-infected GR-deficient RBCs from the index patient, brother (individual 2) and sister (individual 3) of the index patient, and from normal GR-sufficient controls. Phagocytosis by human monocytes (panel B) of ring- and trophozoite-infected and non-infected GR-deficient RBCs from the index patient, brother (individual 2) of the index patient, and from normal GR-sufficient controls. Black bars indicate ring-/trophozoite-infected RBCs; open bars, non-infected RBCs. Phagocytosis is expressed as the number of ingested RBCs per phagocyte. Representative data of index patient, individuals 2 and 3, and mean values of normal controls (mean±SD, n = 3) are shown. For experimental details see Materials and Methods.

Membrane binding of complement fragments C3 and C3b_2_ and autologous IgG, two powerful opsonins known to be instrumental in inducing phagocytosis, were assayed in ring-infected RBCs in the index patient as well as in individuals 2 and 3. Opsonic complement C3 fragments C3b/C3b_2_ were measured as C3c, a stable C3 derivative, making use of anti-C3c antibodies, as detailed in the Materials and Methods section. As shown in Figure 1B–C, membrane deposition of complement fragment C3c was remarkably increased in all three GR-deficient individuals, while binding of IgG was strongly enhanced in the index patient but unchanged in the other two individuals. In general, changes observed in the index patient were more pronounced compared to the other two GR-deficient individuals. Phagocytosis was assayed with the human monocytic cell line THP-1 (Figure 2A) and with human mononuclear cells (Figure 2B). Both phagocytic cell types displayed increased phagocytosis of ring-infected cells from all three GR-deficient individuals. Again the effect was more pronounced in the index patient. Co-cultivated, non-infected normal or deficient RBCs were not phagocytosed in significant amounts (Figure 2A–B). Trophozoites were phagocytosed more intensely than ring stages, with minor differences between GR-deficient and control RBCs (Figure 2B).

The data on membrane-bound hemichromes, complement C3c fragment, IgG, and phagocytosis were obtained from single samples from the GR-deficient individuals and 3–4 normal controls. Except for hemichromes where 3–4 repeats were constantly performed and formal statistical treatment was possible, the paucity of sample material did not allow us to perform a sufficient number of repeats in the GR-deficient samples. Therefore representative values are shown in Figures 1 and 2. The supplementary figure S1 compares results shown in Figures 1 and 2 with an additional series of normal control rings obtained using 24–36 h old normal blood from 7 healthy Italian donors. In general, SDs were distinctly lower in the Italian control series, increasing the robustness of comparisons.

## Discussion

### RBC mutations leading to enhanced ring phagocytosis

A relatively small number of mutations affecting hemoglobins and RBC enzymes are present in large numbers in human populations. It is accepted that these widespread polymorphisms afford protection against *falciparum* malaria [24]–[26]. A group of these conditions including sickle cell anemia, β-thalassemia, possibly hemoglobins C [27], [28] and E [29], as well as G6PD deficiency [14], [20] and PK deficiency [23] appear to provide antimalarial protection based on a common mechanism [20], [22]. The aberrant RBCs are characterized by an increased production of reactive oxygen species (ROS) (hemoglobin mutants), or decreased antioxidant defense (G6PD deficiency, PK deficiency [23]). Non-infected, mutant RBCs display slight but significantly increased membrane deposition of hemichromes accompanied by low-grade but distinct enhancement of phagocytic uptake [20], [22], [23]. Parasites growing in any of the aforementioned mutated cells induce typical modifications in the host RBCs which start manifesting at ring stage. Normal RBCs harboring ring stages show little band 3 aggregation, poor opsonization, and slightly enhanced phagocytic recognition and uptake [30]. By contrast, rings growing in mutant RBCs sequentially display (1) remarkably enhanced hemichrome deposition on the membrane, (2) oxidative band 3 aggregation, (3) increased affinity for naturally circulating IgG – notably anti-(band 3) IgG, (4) activation of the complement system and generation of C3 opsonic fragments, (5) opsonization by increased deposition of autologous IgG and C3 opsonic fragments, and finally (6) remarkably enhanced recognition and phagocytosis by monocytes/macrophages [20]–[23]. This sequence of events, eventually resulting in enhanced phagocytic uptake, is very similar to changes occurring in the normal human RBC during the very last phase of its lifespan of 120±4 days [21].

### Advantages of enhanced ring phagocytosis for the malaria patient

Enhanced and preferential phagocytosis of ring-parasitized mutant RBCs may be advantageous to the host in two ways: (i) *via* reduction of parasite growth and parasite density, observed for example in patients with HbAS and β-thalassemia trait [24], [25], and (ii) *via* rapid digestion of phagocytosed rings by monocytes and the frequent repetition of this process without loss of efficiency [31]. By contrast, phagocytosis of hemozoin-containing mature parasites inhibits the ability of monocytes to repeat the phagocytic process [31], enhances their production of inflammatory cytokines [32], and impairs their ability to kill ingested pathogens [33], to express MHC class II and other membrane antigens upon interferon-gamma stimulation [34], and to correctly present antigens [35]. Another way enhanced ring phagocytosis may be advantageous to the host is by lowering the number of trophozoites and schizonts adhering to endothelia in specific organs and provoking there severe clinical conditions such as cerebral malaria, placental malaria, and possibly dyserythropoiesis-based anemia [36], [37]. Other protection mechanisms probably concur with this based on enhanced ring-phagocytosis of mutant RBCs underscored here. For example, decreased adhesion to endothelia has been suggested to be involved in anti-malaria defense in sickle-cell trait [38], [39] and Hb AC/CC [40].

### ApoGR-deficient red blood cells as host cells for P. falciparum

GR deficiency and G6PD deficiency might show similar characteristics as far as instability and impaired regeneration of GSH from GSSG is concerned [41], [17]. However, G6PD deficiency may have more severe functional consequences for the RBC than GR deficiency, since insufficient formation of NADPH leads to an inadequate supply of reducing equivalents to both the glutathione and the thioredoxin system, whereas the absence of GR activity in non-parasitized RBCs is obviously well compensated [18]. As indicated already by previous studies, rather stable GSH concentrations are present in the non-parasitized RBCs [17]. This might be due to enhanced GSH synthesis [17] but could also be the result of upregulation of the pentose phosphate shunt and by components of the thioredoxin system. Also in the host-parasite unit, the absence of RBC GR seems to be well tolerated under cell culture conditions and in the absence of oxidative stressors. This is shown by normal invasion and growth as well as by comparable glutathione levels detected in both parasites grown in GR-deficient and normal RBCs. Interestingly, GR activity was found to be downregulated by 40% in the parasite compartment. The observation that this decrease is not reflected in the glutathione levels can be explained by the fact that GR is working intracellularly with large spare capacity [9, and references therein]. This phenomenon, however, needs to be studied in further detail. An efficient adaptation to the lack of host cell GR in our *in vitro* system was furthermore supported by the fact that the IC_50_ values for CQ, artemisinin, and methylene blue were very similar when comparing parasites grown in GR-deficient RBCs and in GR-sufficient control RBCs. All three antimalarial drugs are known to interfere with redox metabolism: CQ inhibits heme polymerization, leading to enhanced oxidative stress; artemisinin as an endoperoxide is able to increase ROS concentrations; methylene blue is a known redox cycler and *Pf*GR inhibitor [4], [5]. Only when incubating parasitized RBCs with micromolar concentrations of paraquat [42] or of the nitric oxide releasing agent sodium nitroprusside, IC_50_ values were significantly lower in parasites grown in GR-deficient RBCs than in the controls. This result suggests that it might be possible to demonstrate the higher redox susceptibility of GR-deficient parasitized RBCs in an *in vitro* assay by imposing oxidative or nitrosative stress.

### Enhanced ring phagocytosis associated with GR deficiency

Data presented here show that malaria-infected GR-deficient RBCs behave very similarly to infected G6PD-deficient RBCs [20]. First, GR-deficient RBCs were invaded by parasites, and they supported the growth and multiplication of CQ-sensitive and -resistant *P. falciparum* strains in a manner similar to normal RBCs. Second, as expected from the enhanced sensitivity to oxidation, infection enhanced heme and hemichrome deposition in deficient RBCs by a factor of approximately 3. Third, membrane deposition of opsonins such as C3 complement fragments and autologous IgG, and phagocytosis, were also found to be increased when ring stages were grown in GR-deficient RBCs. The two other homozygous GR-deficient individuals examined here (brother and sister of the index patient) also showed enhanced binding of C3 complement fragment and increased phagocytosis whereas IgG binding was not increased. These differences remain to be elucidated in further detail. All three individuals studied were identical with respect to the complete lack of GR activity. However, other metabolic or immunologic states and predispositions, which we do not know and therefore cannot control, might lead to the observed differences.

### Endemic GR deficiency caused by undersaturation with FAD

Complete absence of RBC GR is very rare [12], [17], [18]. This defect evidently did not arise in parallel to malaria expansion in humans to provide protection. However, impairment of GR activity is widespread and geographically coincident with past or present occurrence of malaria. Since GR is a flavoprotein critically dependent on FAD [43], flavin-deficient RBCs would contain decreased GR activity. In fact, riboflavin deficiency is widespread in underdeveloped countries due to low dietary intake [44], [45]. Several studies have shown that a reduced riboflavin status and non-genetic low GR activity are associated with lower *Plasmodium* parasitemia in humans [46] and in animal [47] models and are frequently found in malaria-endemic areas [48]. Furthermore, high prevalence of familial flavin-deficient RBCs not due to dietary riboflavin deficiency was detected in parts of Italy where malaria used to be prevalent, namely the Maremma region in Tuscany, the delta region of the river Po, and coastal areas in Sardinia [48]–[50]. Interestingly, in Sardinia [50] micro-regional accumulation in villages formerly exposed to malaria was observed for carriers of the putative malaria-protective low-activity GR and FMN-dependent pyridoxine phosphate oxidase [50]. Accumulation in the same villages was previously observed for carriers of β-thalassemia and G6PD deficiency [51]. Finally, low GR activity was found to be frequently associated with other malaria-protective mutations such as sickle-cell anemia and β-thalassemia [52]–[55].

### Conclusions

In conclusion, rare RBC mutations such as PK deficiency and GR deficiency resist malaria infection and may provide protection by the same paradigm applicable to widespread mutations such as those affecting hemoglobin, G6PD, or membrane proteins. Thus the pharmacologic inhibition of parasite and/or host cell GR might lead to metabolic consequences proven by nature to be effective against the parasite. Indeed the inhibition of RBC GR has been proposed as a feasible antimalarial approach likely to reduce the risk of resistance development [9]. Normal RBCs pretreated with high doses of the cytostatic agent carmustine (BCNU) have no detectable GR activity. These RBCs do not serve as host cells of *P. falciparum in vitro* unless the medium contains high levels of glutathione. Thus, in contrast to genetically GR-deficient RBCs, RBCs rendered GR-deficient by BCNU appear to be unable to maintain sufficient GSH levels, possibly because the high BCNU doses also affect the GSH synthesizing enzymes [56]. Nevertheless erythrocytes pretreated with lower doses of nitrosoureas *in vivo* or *in vitro* are probably suitable for corroborating and extending the findings of this present study [57].

When testing GR-inhibitors as antiplasmodial agents it should be taken into account that the effects on parasite growth and multiplication *in vitro* might be less pronounced than *in vivo* – as observed in genetic GR deficiency. Thus additional tests such as parallel application of oxidant stressors and/or determination of membrane alterations and phagocytosis should be performed for assessing the antimalarial effects of GR inhibitors. Furthermore their hemolytic potential needs to be evaluated carefully.

## Materials and Methods

### Patients

The GR-deficient patient – the index patient – who kindly agreed to donate blood for this study was a 54-year old woman whose clinical history has been described before [17], [18]. She was the index patient in a family with GR deficiency who suffered a hemolytic crisis after eating fava beans. Two siblings – brother and sister of the index patient, indicated as individual 2 and individual 3, respectively – were also homozygous for GR deficiency [17]. In all three individuals GR activity in RBCs and leukocytes was undetectable and could not be stimulated by either riboflavin ingestion *in vivo* or FAD addition *in vitro* [18].

All family members included in this study are fully aware of their unique enzyme deficiency and have given their written consent for the research carried out. The research has been explained to them both in written and oral form. The research was performed as part of the diagnostic work on the patients' blood samples, which is fully approved by the Sanquin Ethical Medical Committee.

### GR activity in GR-deficient, non-infected RBCs and GR-deficient leukocytes

As previously reported, the GR activity in the patients' non-infected RBCs was below the detection limit of our assay, while leukocytes had 15% of normal activity. However, taking into account the basal NADPH oxidation rate measured in the absence of GSSG (14–63 µmoles/min/10^11^ cells at 25°C), only minimal GR activity was present in patient's or her siblings' leukocytes (<5 µmoles/min/10^11^ cells at 25°C) [18]. Furthermore neither non-infected RBCs nor leukocytes of the patient or her siblings contained detectable amounts of GR protein. In direct comparison, healthy adult control persons (n = 100) had GR activities of 2.7–5.8 IU/g hemoglobin in RBCs and 118–164 IU/10^11^ leukocytes [18].

GSH levels were already measured and reported for the GR-deficient patients in 1976. GSH was measured in the red cells of all family members and found to be normal, with values of 56–63 mg of GSH per 100 ml of packed red cells [17].

### Preparation and shipment of blood samples

For the studies performed in Giessen (infection by 3D7 and K1 *P. falciparum* strains, parasite cultivation, synchronization, invasion and multiplication studies, determination of glutathione and enzyme activities in parasites, drug sensitivity tests), EDTA-blood samples were taken from the patients and from hematologically healthy control persons in the Netherlands, immediately shipped to Germany on crushed ice, and utilized within 24 h. For the studies performed in Turin (infection by the Palo Alto *P. falciparum* strain and parasite cultivation, invasion and multiplication studies, synchronization of parasite cultures and stage-dependent separation of infected RBCs, quantification of membrane-bound total heme, hemichromes, IgG and complement C3c fragment, assay of phagocytosis with stage-separated infected and control RBCs), full blood from the GR-deficient individuals and three normal controls was anticoagulated with CPD, shipped on crushed ice, and utilized within 24 h of venipuncture. The further processing of the blood samples is described below.

### Cultivation of P. falciparum and preparation of parasite extracts

In the Giessen lab CQ sensitive (3D7-Netherlands) and resistant (K1-Southeast Asia) strains of *P. falciparum* were grown in continuous culture as described [58] with slight modifications. Parasites were maintained at 1–10% parasitemia and 3.3% hematocrit in RPMI 1640 culture medium supplemented with A^+^ RBCs (normal culture conditions) or 0^+^ RBCs (for the GR-deficient patient and a control sample), 4% A^+^ human serum, 0.2% lipid-rich bovine serum albumin (Albumax), 9 mM glucose, 0.2 mM hypoxanthine, 2.1 mM L-glutamine, and 22 µg/ml gentamicin. All incubations were carried out at 37°C, 3% O_2_, 3% CO_2_ and 94% N_2_. Synchronization of parasites in culture to ring stages was carried out by treatment with 5% (w/v) sorbitol [59]. The morphology of the parasites as well as the multiplication rate were determined by light microscopy after Giemsa staining. Before determining either the glutathione content or the GR activity in *P. falciparum*, parasites were grown over 5 cycles (10 days) in their respective RBCs. Trophozoite stage parasites were then isolated by suspending the RBCs in a 20-fold volume of buffer containing 7 mM K_2_HPO_4_, 1 mM NaH_2_PO_4_, 11 mM NaHCO_3_, 58 mM KCl, 56 mM NaCl, 1 mM MgCl_2_, 14 mM glucose, and 0.02% saponin for 10 min at 37°C. The pellets were washed two times in the same saponin buffer for RBC lysis and one time in PBS (centrifugation for all steps 1,500 g, 5 min, room temperature). The free parasites were finally diluted in PBS and disrupted by freezing and thawing three times in the presence of protease inhibitors (40 µl/ml Complete (Roche) and 1 mg/ml Pefabloc (Roche)). After centrifugation (ultracentrifuge 100,000 g, 30 min, 4°C), the protein content of the supernatant was determined by the Bradford method and the extract was used for the various analyses.

### Invasion and multiplication of P. falciparum grown in GR-deficient RBCs

To determine if *P. falciparum* can successfully invade and multiply in GR-deficient RBCs, EDTA full blood was taken from patients and controls in the Netherlands and shipped at 4°C to the Giessen lab. About 24 h after venipuncture, plasma and buffy coat of the blood samples were removed by centrifugation (1,500 g) and aspiration followed by washing three times in a 10-fold volume of RPMI 1640 medium (1,500 g, 3 min, 4°C). For some of the invasion studies control samples freshly taken in Germany were processed and studied in parallel.

EXPERIMENT 1: Ten µl of RBCs from the index patient (0^+^ blood) and two German controls (A^+^ and 0^+^ blood) each were added to 300 µl of complete cell culture medium in 48-well plates. Two µl of parasitized RBCs (3D7, mainly trophozoite stage; final parasitemia 0.7%) were added to the RBC cultures. The K1 strain was inoculated for drug sensitivity tests.

EXPERIMENT 2: In a separate experiment, RBCs from the index patient (0^+^ blood) were shipped together with 3 controls (0^+^, A^+^, A^+^ blood). After washing, 0.5 ml of RBCs were added to 15 ml of cell culture medium and inoculated with 1 ml of parasitized RBCs (3D7, synchronized to ring stages; this resulted in the same final parasitemia of 0.7% as in experiment 1).

### Determination of total glutathione and PfGR activity in parasite extracts

For the determination of total glutathione content, 40 µl of parasite extract was deproteinized by adding 2 vol of 5% (w/vol) sulfosalicylic acid; the samples were mixed and centrifuged, and the supernatant was used for analyses. The glutathione content was measured by the GR-coupled 5,5′-dithio-bis(2-nitrobenzoic acid, DTNB)-GSH-recycling assay [60]. A standard curve was prepared using appropriate concentrations of GSH and sulfosalicylic acid. *P. falciparum* (*Pf*)GR activity in the parasite extracts was assayed at 25°C with 100 µM NADPH and 1 mM GSSG in 47 mM potassium phosphate, 200 mM KCl, 1 mM EDTA, pH 6.9. The consumption of NADPH was followed spectrophotometrically at 340 nm [61]. Specific activities (µmol/min/mg of protein) were calculated using the enzyme activities at 25°C and the protein concentrations of the parasite extracts.

### Drug effects on P. falciparum

An isotopic drug sensitivity assay using the semi-automated microdilution technique [62] was employed to investigate the effects of CQ, artemisinin, methylene blue, paraquat and sodium nitroprusside (SNP) on parasites grown in GR-deficient RBCs. The method is based on the incorporation of radioactive ^3^H-hypoxanthine – which is taken up by the parasite as a precursor of purine deoxynucleotides for DNA and RNA synthesis – and was modified according to Fivelman [63]. In 96-well microtiter plates (Nunc^R^), a two-fold serial dilution of the starting concentration of each drug to be tested was carried out. Parasites were incubated at a parasitemia of 0.125% (>70% ring forms) and 1.25% hematocrit in hypoxanthine-free medium. After 48 h, 0.5 µCi ^3^H-hypoxanthine was added into each well and the plates were incubated for another 24 h. The cells from each well were harvested on a glass fiber filter (Perkin-Elmer, Rodgau-Jügesheim, Germany), washed, and dried. Their radioactivity in counts per min was considered to be proportional to the respective growth of *P. falciparum* in the well. IC_50_ values (drug concentrations that produce 50% reduction in the uptake of ^3^H-hypoxanthine) were calculated as described in [63].

For statistical analyses of the data on multiplication rates, biochemical parameters and IC_50_ values (given as mean values ± SEM), the unpaired t-test as well as the Mann-Whitney U Test were employed.

### Stage-dependent separation and opsonization of parasites

*P. falciparum* parasites (Palo Alto strain, *Mycoplasma-*free) were cultivated in normal and GR- deficient RBCs at 2% hematocrit and synchronized as described [30]. Briefly, schizont stage infected normal RBCs (parasitemia >95%) were mixed for invasion with washed GR-deficient or normal RBCs and kept in growth medium (RPMI 1640, containing 25 mM Hepes, 30 mM glucose, 2 mM glutamine, 0.02 mM adenine, 24 mM NaHCO_3_, 32 mg/l gentamicin, and 10% A^+^ decomplemented human plasma) (time 0). After 19 h incubation in a humidified CO_2_/air incubator, the ring-enriched fraction was separated on and collected from a discontinuous 40/80/90% Percoll gradient, containing mannitol (6% wt/vol). The bottom Percoll fraction contained non-infected RBCs and approximately 15–20% rings, morphologically identical to those of the ring-enriched fraction. After 41 h incubation the trophozoite-enriched fraction was separated onto and collected from a discontinuous 10/40/80% Percoll gradient. Rings (after 19 h incubation) as well as trophozoites (after 41 h incubation) were enriched to approx. 80–85% and approx. 90%, respectively, by the above procedure. Parasitemia and parasite morphology were assessed by light microscopy after Diff-Quik® Fix staining (Medion Diagnostics GmbH, Düdingen, Switzerland). Non-infected control RBCs of each donor were incubated and treated in a similar way without schizont inoculation at time 0. Infected and non-infected RBCs were washed, rejuvenated for 1 h in PBS-G, and opsonized with freshly drawn serum of a healthy AB/Rh^+^-donor in a ratio: RBCs/PBS containing 2 mM glucose (PBS-G)/serum 1/1/2 (vol/vol). Non-opsonized RBCs were incubated in parallel substituting serum with PBS-G. After 30 min at 37°C cells (ring-enriched, trophozoite-enriched or non-infected RBCs) were washed 3 times with PBS-G, adjusted to 50% hematocrit and analyzed by FACS for surface-bound IgG and complement C3c fragment, and utilized for the phagocytosis assays with THP-1 cells and human peripheral monocytes. Total heme and hemichromes were measured in hypotonic ghosts prepared from the bottom Percoll fraction of the unseparated, infected GR-deficient and normal RBCs (see below). The latter procedure was due to the paucity of the ring material obtained by the enriched ring fraction (approx. 30–40 µl per individual) sufficient to perform cytofluorimetric studies but not sufficient for ghost preparation.

### Quantification of membrane-bound total heme and hemichromes in ghosts prepared from stage-separated infected RBCs

Heme bound to the cytoplasmic face of the membrane as hemoglobin and insoluble hemichromes was analyzed in the hypotonic ghosts [64] prepared under non-reducing conditions in the presence of Complete® protease inhibitor cocktail from the bottom Percoll fraction of the unseparated, infected GR-deficient and normal RBCs [22]. This fraction contained approximately 15–20% rings. Every ghost preparation was divided into two aliquots. A first aliquot of 10 µl ghosts was solubilized for 3 h at 4°C in 1 ml of 0.1 N NaOH containing 3 mM EDTA and 0.05% (vol/vol) Triton X100 to quantify the total heme content (hemoglobin + insoluble hemichromes). A second aliquot of 10 µl ghosts was solubilized for 1 h at room temperature in 1 ml of PBS containing 3 mM EDTA and 0.05% (vol/vol) Triton X100 and used to quantify hemoglobin. Heme from both ghost aliquots was quantified by measuring the heme-dependent luminol-enhanced luminescence as described [65]. Each luminescence measurement (2 µL aliquots) was repeated 3–4 times. Inter-measurement variability never exceeded 2–3%. Results obtained from cell samples that contained approx. 15–20% rings were extrapolated to 100% rings using the calculation suggested by Cappadoro *et al.* [20]. The hemichrome content was calculated from the difference between total heme and hemoglobin heme. Hemichrome heme was the major component of total heme in control rings (88% of total heme), index patient (96%), individual 2 (89%) and individual 3 (87%), while membrane-bound hemoglobin was a minor fraction of total heme.

### Quantification of complement C3c fragment and IgG by flow cytometry

Ten million ring- or trophozoite-infected and non-infected normal or GR-deficient RBCs were incubated for 30 min at room temperature in 50 µl of PBS-G, with or without the primary rabbit anti-human complement C3c fragment antibody or anti-human IgG antibody (Sigma, Milano, Italy), at a 1∶400 dilution, respectively. Complement C3c fragment is the stable derivative of C3b/C3b_2_ opsonins [66]. Thereafter infected RBCs and non-infected controls were washed twice, suspended in 50 µl PBS-G, and incubated for 30 min at room temperature with anti-rabbit IgG-FITC antibody (goat) at 1∶400. At the end of the incubation, cells were washed twice with 200 µl PBS-G, suspended in PBS-G, and analyzed on a BD FACSCalibur Flow Cytometer using Cell Quest software (BD Biosciences). Events were displayed on green fluorescence (FL1) versus forward scatter (FSC) dot plots or FL1 versus events number histogram. RBCs were gated on light scatter characteristics, and a total of at least 30,000 events in gating were collected for each sample. Data analysis was done with WinMDI software.

### Phagocytosis of stage-separated infected and control RBCs by THP-1 cells and human mononuclear cells

The phagocytosis assay was performed utilizing the human monocytic cell line THP-1 or human mononuclear cells.

1. THP-1 cells were maintained at 0.2×10^6^ cells/ml in RPMI 1640 medium supplemented with 10% heat-inactivated fetal bovine serum (FBS), 2 mM L-glutamine, 100 U/ml penicillin, and 100 µg/ml streptomycin. The phagocytic ability of THP-1 cells was stimulated by supplementing IFN-gamma (50 U/ml) and TNF (250 U/ml) to the cell suspension 30 h before the start of phagocytosis. Shortly before phagocytosis, cells were washed three times with RPMI 1640 medium and resuspended in Macrophage-SFM medium supplemented with 10% heat-inactivated FBS to obtain 150,000 cells/300 µl medium. Ring- or trophozoite-infected and non-infected normal or GR-deficient RBCs were fluorescent-labeled for the phagocytosis assay during 10 min incubation at room temperature with 0.5 µM carboxyfluorescein diacetate, succinimidyl ester, (CFDA-SE, Sigma, Milano, Italy) in PBS-G at 0.05% hematocrit. Labeling was stopped by adding 2 ml of heat-inactivated FBS for 5 min. RBCs were then washed three times with PBS-G and co-incubated with THP-1 cells at a THP-1:RBC- ratio of 1∶100 in round-bottom polypropylene tubes at 37°C for 2.5 h (CO_2_ 5%). At the end of the incubation, non-ingested RBCs were lysed with 3.5 ml of ice-cold distilled water for 2 min. Physiologic osmolarity was restored by adding 10-times concentrated PBS. Cells were finally washed with cold PBS +1% FBS and analyzed with a BD FACSCalibur Flow Cytometer using Cell Quest software (BD Biosciences). Events were displayed on green fluorescence (FL1) versus forward scatter (FSC) dot plots or FL1 versus events number histogram. THP-1 live cells were gated on light scatter characteristics, and a total of at least 30,000 events in gating were collected for each sample. Data analysis was done with WinMDI software.

2. Human mononuclear cells were separated by Ficoll separation from buffy coats of healthy donors freshly discarded from the local blood bank, suspended in Macrophage-SFM medium (Invitrogen, Carlsbad, CA), and adhered to 24-well cell culture plates as described [67]. Phagocytosis was started by the addition of 100 opsonized ring- or trophozoite-infected or non-infected normal or GR-deficient RBCs per monocyte, stopped after 3 h incubation in the CO_2_-incubator, and non-ingested RBCs were removed by lysis and 4 washing steps. Phagocytosis was quantified by measuring the heme-dependent luminol-enhanced luminescence of ingested hemoglobin-heme as described [65].

## Supporting Information

Figure S1 Membrane-bound hemichromes, phagocytosis, complement C3c fragment, and autologous IgG in/of GR-deficient and GR-sufficient RBCs - comparison with healthy Italian donors. Data from Figure 1 and Figure 2 of the manuscript were compared to ring-infected and non-infected GR-sufficient control RBCs prepared from 24–36 h old blood (kept at +4°C) from 7 healthy Italian donors. Mean values of normal controls (mean±SD, n = 7).
(1.65 MB TIF)
