# Supplementary figures and images for: Inherited Glutathione Reductase Deficiency and Plasmodium falciparum Malaria—A Case Study
Source: PLoS One. 2009 Oct 6;4(10):e7303. doi: 10.1371/journal.pone.0007303 (PMC2751828; doi:10.1371/journal.pone.0007303)

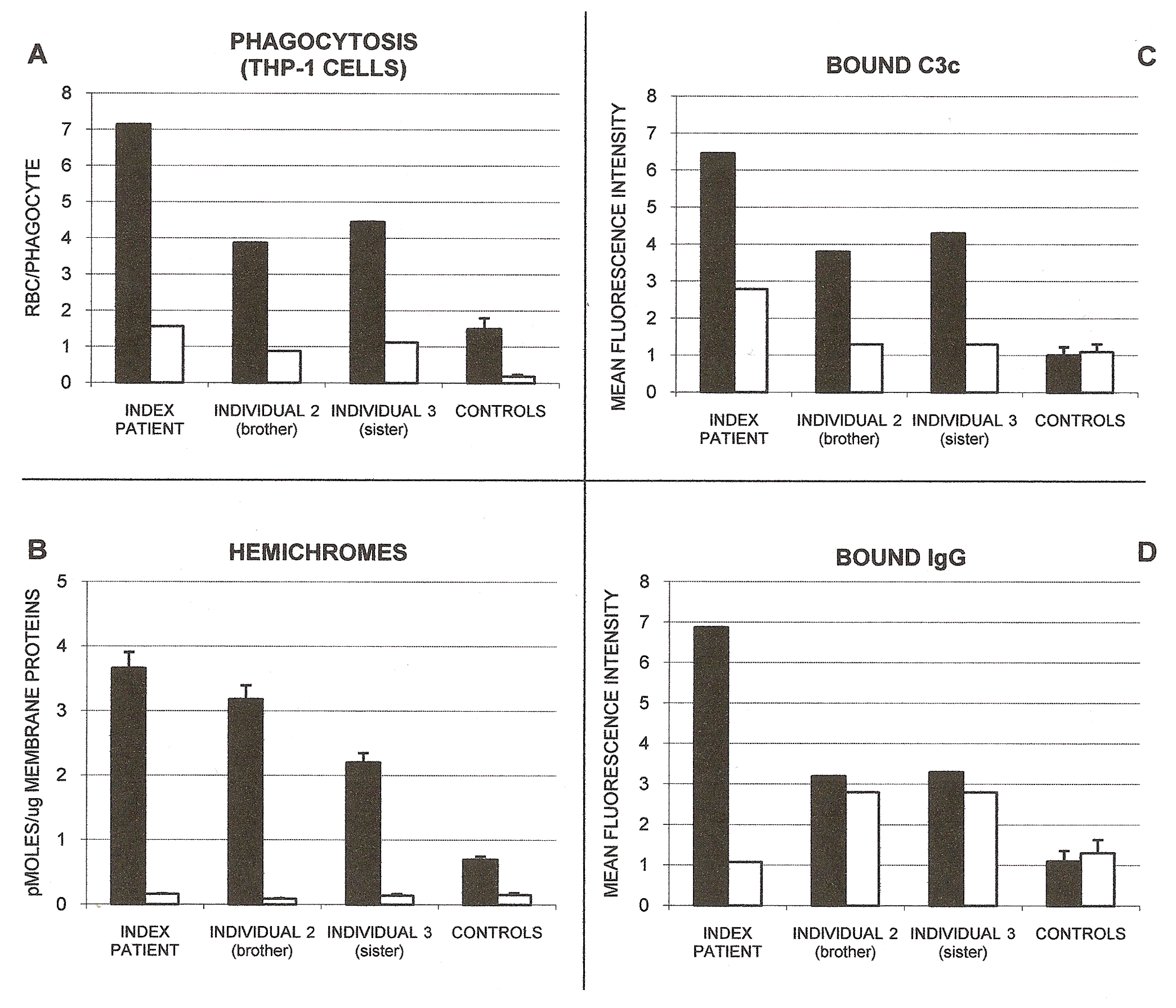

Supplement: Figure S1 — Membrane-bound hemichromes, phagocytosis, complement C3c fragment, and autologous IgG in/of GR-deficient and GR-sufficient RBCs - comparison with healthy Italian donors. Data from Figure 1 and Figure 2 of the manuscript were compared to ring-infected and non-infected GR-sufficient control RBCs prepared from 24–36 h old blood (kept at +4°C) from 7 healthy Italian donors. Mean values of normal controls (mean±SD, n = 7). (1.65 MB TIF) [file pone.0007303.s001.tif]
